# Co-Encapsulation of Simvastatin and Doxorubicin into pH-Sensitive Liposomes Enhances Antitumoral Activity in Breast Cancer Cell Lines

**DOI:** 10.3390/pharmaceutics15020369

**Published:** 2023-01-21

**Authors:** Jaqueline Aparecida Duarte, Eliza Rocha Gomes, André Luis Branco De Barros, Elaine Amaral Leite

**Affiliations:** 1 Department of Pharmaceutical Products, Faculty of Pharmacy, Federal University of Minas Gerais, Av. Antônio Carlos, 6627, Belo Horizonte 31270-901, Brazil; 2 Department of Clinical and Toxicological Analyses, Faculty of Pharmacy, Federal University of Minas Gerais, Av. Antônio Carlos, 6627, Belo Horizonte 31270-901, Brazil

**Keywords:** breast cancer, pH-sensitive liposomes, coencapsulation, doxorubicin, simvastatin

## Abstract

Doxorubicin (DOX) is a potent chemotherapeutic drug used as the first line in breast cancer treatment; however, cardiotoxicity is the main drawback of the therapy. Preclinical studies evidenced that the association of simvastatin (SIM) with DOX leads to a better prognosis with reduced side effects and deaths. In this work, a novel pH-sensitive liposomal formulation capable of co-encapsulating DOX and SIM at different molar ratios was investigated for its potential in breast tumor treatment. Studies on physicochemical characterization of the liposomal formulations were carried out. The cytotoxic effects of DOX, SIM, and their combinations at different molar ratios (1:1; 1:2 and 2:1), free or co-encapsulated into pH-sensitive liposomes, were evaluated against three human breast cancer cell lines (MDA-MB-231, MCF-7, and SK-BR-3). Experimental protocols included cell viability, combination index, nuclear morphological changes, and migration capacity. The formulations showed a mean diameter of less than 200 nm, with a polydispersity index lower than 0.3. The encapsulation content was ~100% and ~70% for DOX and SIM, respectively. A more pronounced inhibitory effect on breast cancer cell lines was observed at a DOX:SIM molar ratio of 2:1 in both free and encapsulated drugs. Furthermore, the 2:1 ratio showed synergistic combination rates for all concentrations of cell inhibition analyzed (50, 75, and 90%). The results demonstrated the promising potential of the co-encapsulated liposome for breast tumor treatment.

## 1. Introduction

Breast cancer was the most common cancer in the world in 2021, and more than 2.2 million cases were diagnosed [1]. Among chemotherapy regimens for treatment of this tumor, anthracyclines, especially doxorubicin (DOX), are the most often used. Despite the expressive clinical response rate, DOX induces serious adverse effects, mainly cardiotoxicity [2]. Furthermore, DOX can induce chemotherapeutic resistance, resulting in poor prognosis and survival of patients. The search for new strategies capable of overcoming these problems has become essential.

Nanomedicine has brought a favorable therapeutic option for DOX; it was the first approved DOX-liposomal formulation. Doxil^®^ (Janssen Biotech, Inc., Johnson & Johnson, Horsham, PA, USA) showed antitumor efficacy comparable to free DOX, and also reduced heart damage [3].

Currently, other DOX-loaded nanoformulations are either approved or in advanced clinical studies for cancer treatment [4,5]. Although many of these products have been proven to be beneficial, with comparable efficacy to free DOX, side effects have still been reported with their use [6,7]. The association of DOX with other antitumor drugs has also been proposed as an alternative to improve therapeutic efficacy; however, this strategy has increased the side effects [8,9].

It has already been pointed out that the SIM increases the cytotoxic effect of DOX in breast cancer cell lines through negative regulation of the cell cycle or induction of apoptosis [10]. Nowadays, studies have investigated combinatorial chemotherapy regimens with compounds from different classes, such as statins, to increase the cytotoxic effect of conventional chemotherapeutic drugs [11]. Preclinical evidence suggests that simvastatin (SIM) has effects beyond its cholesterol-lowering properties, since it may have antitumor effects and decrease cardiac toxicity induced by DOX [12]. A meta-analysis of 1,111,407 cancer patients showed that the administration of SIM reduced cancer mortality by 40% [13]. In this context, the co-encapsulation of these drugs in liposomal formulation could be a promising strategy for improving efficacy and reducing toxicity. In addition, the association of both in the same nanosystem would allow simultaneous delivery to the target site [14]. Recently, a dual-drug liposomal encapsulation of daunorubicin and cytarabine (Vyxeos^®^) in a synergistic 1:5 molar ratio was approved by the Food and Drug Administration and European Medicines Agency for myeloid leukemia treatment [15].

Our group has developed pH-sensitive liposomes for carrying DOX, and the rationale behind the proposal is based on (i) the acidic pH of the tumor microenvironment and intracellular endosomes (around 6.5 and 5.0–6.0, respectively) compared to normal tissues (pH 7.4); (ii) presence of a polymorphic lipid capable of forming a lamellar bilayer at physiological pH as well as undergoing destabilization and change to a hexagonal phase, releasing the vesicle content at acidic pH; and (iii) drug circulation time prolonged by using a PEGylated liposome [16].

Herein, the aim was to develop and characterize a pH-sensitive liposomal formulation for co-delivery of DOX and SIM, as well as to evaluate the effect of different ratios of free or encapsulated drugs against human cancer cell lines to investigate their potential for breast cancer treatment.

## 2. Materials and Methods

### 2.1. Materials

1,2-Dioleoyl-sn-glycero-3-phosphoethanolamine (DOPE) and 1,2-distearoyl-sn-glycero-3-phosphoethanolamine-N-[amino(polyethylene glycol)-2000 (DSPE-PEG2000) were supplied by Lipoid GmbH (Ludwigshafen, Germany). Cholesterol hemisuccinate (CHEMS), phosphate-buffered saline (PBS), sodium hydroxide, 4-(2-hydroxyethyl)piperazine-1-ethane sulfonic acid (HEPES), ammonium sulfate, and sodium bicarbonate were obtained from Sigma-Aldrich (St. Louis, MI, USA). Polysorbate 80 (Tween™ 80) was provided by Croda Inc (Edison, NJ, USA). Chloroform and dimethylsulfoxide (DMSO) were provided from Synth (São Paulo, Brazil). Sodium chloride and HPLC-grade methanol were purchased from Merck (Frankfurt, Germany). Doxorubicin hydrochloride (DOX) was purchased from ACIC Chemicals (Brantford, ON, Canada). SIM was acquired from Fagron (São Paulo, Brazil) with a purity greater than 98.0%. The water used in the experiments was purified using the Milli-Q^®^ distillation and deionization equipment (Millipore, MA, USA). The other substances used were of analytical grade.

Human breast tumor cell lines (MDA-MB-231, MCF-7, SK-BR-3) were purchased by American Type Culture Collection (Manassas, VA, USA). Culture media (Dulbecco’s Modified Eagle’s Medium, DMEM; Minimum Essential Medium, MEM and McCoy), Fetal bovine serum (FBS), penicillin, and streptomycin were obtained from Gibco Life Technologies (Carlsbad, USA). Sulforhodamine B (SRB), tris(hydroxymethyl)aminomethane (Tris base), and trypsin were obtained from Sigma-Aldrich (St. Louis, MI, USA) and Hoechst 33258 (Thermo Fisher Scientific—Waltham, MA, USA).

### 2.2. Liposome Preparation

Blank liposomes (SpHL) and SIM-loaded liposomes (SpHL-S) were prepared by the lipid film hydration method. Chloroform aliquots of DOPE, CHEMS, DSPE-PEG2000 (molar ratio 5.8:3.7:0.5, respectively; total lipid concentration of 20 mM) were transferred to a round bottom flask. For SpHL-S, SIM chloroform solution (concentration 1 mg/mL) was added to lipids. The solvent was removed under reduced pressure using a Buchi Labortechnik AG Rotator CH-9233, model R-210, coupled to a V-700 vacuum pump (Flawil, Switzerland). After total solvent evaporation, a solution of NaOH in molar ratio NaOH:CHEMS, equal to 1, was added to the lipid films, followed by the addition of ammonium sulfate solution (300 mM), pH 7.4, under vigorous agitation. The unencapsulated SIM was then separated from the liposomes by centrifugation at 3000 rpm, 25 °C, for 10 min (Heraeus Multifuge X1R centrifuge, Thermo Fischer Scientific, Waltham, MA, USA).

The diameter of the vesicles was calibrated by the ultrasound (model CPX 500; 500 W, Cole-Parmer Instruments, Vernon Hills, IL, USA) using a Stepped microtip S&M 630-0418 nail with 21% amplitude for 5 min in an ice bath. The preparations were purified to eliminate external ammonium sulfate by ultracentrifugation (Ultracentrifuge Optima^®^ L-80XP, Beckman Coulter, Brea, CA, USA) at 150,000× *g* and 4 °C for 120 min. The pellets were resuspended with 0.9% (*w*/*v*) NaCl solution, maintaining the initial lipid concentration.

For the preparation of liposomes containing DOX, freshly prepared liposomes (SpHL or SpHL-SIM) were incubated with DOX solution (1 or 2 mg/mL) for 2 h at 4 °C, and the encapsulation was performed by remote loading driven by a transmembrane sulfate gradient to obtain the final dispersion of SpHL-D and SpHL-D-S, respectively. Non-encapsulated DOX was removed by ultracentrifugation at the same conditions previously described (Ultracentrifuge Optima^®^ L-80XP, Beckman Coulter, Brea, CA, USA). The purified pellet was resuspended with saline.

In order to guarantee the molar ratios in the liposomal form used in vitro studies, after quantifying SIM concentration, DOX was loaded, driven by the transmembrane ammonium sulfate gradient. As the DOX encapsulation efficiency is approximately 100%, DOX: SIM molar ratios 1:1, 1:2, or 2:1 were ensured.

### 2.3. Physicochemical Characterization

#### Mean Diameter, Polydispersity Index (PDI), and Zeta Potential

The mean diameter of the vesicles and the polydispersity index (PDI) were determined by dynamic light scattering (DLS) at 25 °C and a fixed angle of 90°. The zeta potential was determined by the electrophoretic mobility associated with DLS. All samples were diluted in 0.9% NaCl solution (*w*/*v*) at a ratio of 1:100 and measured, in triplicate, using Zetasizer NanoZS90 equipment (Malvern Instruments, Worcestershire, UK).

### 2.4. Determination of DOX and SIM Content

DOX and SIM quantification was carried out by high-performance liquid chromatography (HPLC). For DOX determination, the experimental conditions were the same as previously described [17].

SIM quantification was performed with the Agilent 1260 Infinity instrument (Santa Clara, CA, USA) using a mobile phase composed of methanol: 0.1% phosphoric acid solution (90:10 *v*/*v*) and a C18 reversed-phase column of 25 cm × 4.6 mm with a particle size of 5 μm (LichroCar, Merck, Frankfurt, Germany). An injection volume of 10 μL, a flow rate of 1.0 mL/min, and detection with a Diode Array detector G4212B (Santa Clara, CA, USA) at a wavelength of 238 nm and room temperature were used [18].

The liposome sample preparation consisted of the rupture of the lipid membrane with methanol 1:5 *v*/*v,* followed by dilution in the respective mobile phase. Liposomal formulations were quantified before (non-purified liposomes) and after (purified liposomes) purification, and the encapsulation percentage (EP) was calculated according to the following equation:

$$\mathrm{Drug}\;\mathrm{encapsulation}\;\mathrm{percentage}\;\left(\%\right)=\frac{\left[\mathrm{drug}\right]\;\mathrm{in}\;\mathrm{purified}\;\mathrm{liposomes}\;}{\left[\mathrm{drug}\right]\;\mathrm{in}\;\mathrm{non}\;\mathrm{purified}\;\mathrm{liposomes}}\times 100$$

### 2.5. Cryo-Transmission Electron Microscopy

SpHL-D-S images were obtained by cryo-transmission electron microscopy (cryo-TEM) using an FEI Tecnai Spirit G2-12 electron microscope (FEI, Hillsboro, OR, USA) operating at 120 kV. A 3 µL aliquot of the sample was deposited on the previously unloaded carbon grid. The grids were stained with filter paper for 5 s and vitrified by immersion in liquid ethane. The vitrified samples were stored under liquid nitrogen before being transferred to a TEM.

### 2.6. Drug Release Evaluation

The drug release study was carried out at pH 7.4 and 5.0 (corrected by adding 1 mol/L hydrochloric acid). Aliquots of 1 mL of SpHL-D-S (2:1) were transferred to a 10 KDa cut-off cellulose membrane (Sigma, St. Louis, MI, USA) with the ends sealed. The dialysis membrane was placed in an amber bottle containing 100 mL of HEPES buffer plus Tween 80 (0.1% *w*/*v*) to ensure sink condition for both drugs. The flasks were kept under agitation at 156 rpm and 37 °C in an IKA KS 4000i control incubator (Shanghai, China). At each time of investigation (0, 2, 4, 8, 12, and 24 h), a sample was taken and characterized as mean diameter and PDI. In addition, the drug release percentage over time was measured by quantifying DOX and SIM by HPLC, and data were plotted as cumulative percentages of drug release from three independent experiments.

### 2.7. Storage Stability

A freshly prepared liquid dispersion of SpHL-D-S at a molar ratio (DOX:SIM) of 2:1 was kept under a nitrogen atmosphere and protected from light at 4 °C. After 0, 7, 14, 30, 60, and 90 days of preparation, aliquots were collected and the physicochemical characteristics were measured as mentioned above. The mean values were compared with those obtained at day zero.

Storage stability was also evaluated in SpHL-D-S prepared after the reconstitution of lyophilized SpHL-S. In this case, liposomes were prepared as described above, except for the ammonium sulfate remotion step, in which dialysis against HEPES-buffered saline (HBS) pH 7.4 was performed. Then, the formulations were transferred to amber and cryo-resistant flasks containing glucose, as a cryoprotectant, in a sugar:lipid ratio of 2:1 (*w*/*w*). The vials were frozen in liquid nitrogen and lyophilized on a 24 h cycle using a Modulo lyophilizer (Thermo Electron Corporation, Waltham, MA, USA). After the lyophilization cycle, the amber vials were vacuum-sealed and stored at −20 °C. At 0, 7, 14, 30, 60, and 90 days after lyophilization, SpHL-S was reconstituted with ultrapure water and SIM concentration was determined by HPLC. Then, DOX was incubated, as described above, to obtain a DOX:SIM molar ratio of 2:1.

### 2.8. Cell Culture

Human breast adenocarcinoma cells MDA-MB-231 (ATCC HTB-26); MCF-7 (ATCC HTB-22) and SK-BR-3 (ATCC HTB-30) were cultured in DMEM, MEM supplemented with 0.01 mg/mL insulin, and McCoy media, respectively, and all media were supplemented with 10% FBS. Cell lines were cultivated in the presence of penicillin (100 IU/mL) and streptomycin (100 μg/mL) and maintained at 37 °C and 5% CO_2_ in a humidified atmosphere. Prior to the experiments, all cell lines were screened for mycoplasma by polymerase chain reaction (PCR), with negative results.

### 2.9. Cytotoxicity Studies

Tumor cell viability was measured using the sulforhodamine B (SRB) assay. MDA-MB-231, MCF-7, or SK-BR-3 cells were seeded in 96-well plates (1 × 10^4^ cells/well). After 24 h of incubation at 37 °C and 5 % CO_2_, free DOX, free SIM, and free DOX:SIM at molar ratios of 1:1, 1:2, or 2:1, respectively; as well as SpHL-DOX, SpHL-SIM, or SpHL-DOX-SIM at molar ratios of 1:1, 1:2, or 2:1, respectively, were added to the wells (DOX concentration ranged from 0.0195 μM to 40 μM). Free SIM was dissolved in DMSO, and the DMSO concentration for all treatments was less than 1% *v*/*v*. After 48 h of incubation, 10% trichloroacetic acid (TCA) was added to each well in order to fix the cells for 1 h. The plates were then washed with water to remove TCA, followed by staining with SRB for 30 min. Then, the plates were washed with 1% *v*/*v* acetic acid to remove unbound dye. Finally, 10 mM Tris-Base solution (pH 10.5) was added to solubilize the protein-bound dye, and the optical density (OD) was read at 510 nm using a Spectra Max Plus 384 microplate spectrophotometer (Molecular Devices, Sunnyvale, CA, USA).

### 2.10. Determination of the Combination Index (CI)

For the different molar ratios of DOX:SIM, free or encapsulated, the percentage of viable cells was subjected to effect analysis and the combination index (CI) values were determined using CalcuSyn^®^ (Biosoft, Ferguson, MO, USA). CalcuSyn^®^ software allows for the simulation of synergism and antagonism at all dose and effect levels using the median effect algorithm. The values adopted to determine the effects were: synergistic effect, CI < 0.9; additive effect, CI between 0.9 and 1.45; and antagonistic effect, CI > 1.45 [19].

### 2.11. Nuclear Morphometric Analyzes (NMA)

To assess the nuclear morphological changes after treatment, the different cell lines were plated at a density of 2.0 × 10^5^ cells/well in 6-well plates and incubated at 37 °C for 24 h. After incubation, cells were treated with 2 mL of different treatments (DOX, SpHL-D, SIM, SpHL-S, and the mixtures of free DOX:SIM and SpHL-D-S, at a 1:1; 1:2, or 2:1 molar ratio) at a total concentration of 80 nM. After incubation for 48 h, cells were fixed with 4% formaldehyde for 10 min and stained with a Hoescht 33342 (0.2 µg/mL) for another 10 min at room temperature in the dark. Fluorescent images of the nuclei were captured using an AxioVert 25 microscope with a Fluo HBO 50 fluorescence module connected to the Axio Cam MRC camera (Zeiss, Oberkochen, Germany). The analysis was made up of 300 nuclei per treatment using Image J 1.50i Software (National Institutes of Health, Bethesda, CA, USA) and the “NII_Plugin” plugin available at http://www.ufrgs.br/labsinal/NMA/ (accessed on 2 August 2022).

### 2.12. Migration Test

To study the two-dimensional migration, cells were plated at a density of 2.0 × 10^5^ cells/well in 12-well plates and incubated at 37 °C for 24 h. Then, a straight wound was made into individual wells with a 10 µL pipette tip. This point was considered the “zero area” and was imaged using an AxioVert 25 microscope with an Axio Cam MRC camera attached (Zeiss, Oberkochen, Germany).

After obtaining the wounds, the control wells received 1 mL of medium with 1% FBS containing the different treatments (DOX, SIM, and the combination of DOX:SIM at ratios of 1:1; 1:2, or 2:1, respectively, in free or encapsulated form). The drug concentration used for treatment was 80 nM. This represents the total concentration of DOX or SIM alone or a combination of DOX: SIM at different molar ratios in the free or co-encapsulated forms. After 24 h of incubation at 37 °C, cells were fixed with 4% formaldehyde for 10 min. Images along the treated wounds were also obtained in phase contrast. The areas of all wounds were obtained using the MRI Wound Healing Tool plugin for the free version of the Image J 1.45 software (National Institutes of Health, Bethesda, CA, USA). The wound healing percentage was calculated according to the following equation:

$$\mathrm{Wound}\;\mathrm{healing}\left(\%\right)=100-\frac{\mathrm{area}\;\mathrm{of}\;\mathrm{treated}\;\mathrm{wound}\;\times 100}{\mathrm{area}\;\mathrm{of}\;\mathrm{zero}\;\mathrm{wound}}$$

### 2.13. Statistical Analyses

Statistical analyses were performed using GraphPad Software Prism (version 6.00, La Jolla, CA, USA). The normality and homoscedasticity of variance were tested by D’Agostino and Pearson and Brown–Forsythe, respectively. Variables without normal distribution were transformed [log(x + 1)]. The difference between the experimental groups was tested using a one-way analysis of variance (ANOVA), followed by the Tukey test. In vitro studies of nuclear morphology were evaluated two-way, followed by the Bonferroni test. Differences were considered significant when the *p*-value was less than 0.05 (*p* < 0.05). Results were expressed as mean ± SD of at least three independent experiments.

## 3. Results

In this study, we proposed that SIM and DOX be co-encapsulated into pH-sensitive liposomes, aiming to increase antitumor efficacy. Herein, we reported the development and physicochemical characterization of SpHL-D-S, as well as their effects against different human breast cancer cells (MDA-MB-231, MCF-7, and SK-BR-3).

### 3.1. Formulation Development and Physicochemical Characterization

The physicochemical characteristics of the liposomal formulations are summarized in Table 1. All formulations showed an average diameter ranging from 110 to 150 nm, which may allow for the efficient delivery of DOX and SIM to the tumor region due to the EPR effect [20]. In addition, a PDI lower than 0.3 and a zeta potential close to neutrality (−3.0 mV) indicate, respectively, adequate homogeneity and potential for reduced interaction with plasmatic proteins when injected by the intravenous route [21]. There were no significant differences between SpHL and SpHL-D for all parameters evaluated, and the values obtained were consistent with those previously reported [22,23]. Regarding SpHL-S, no significant difference was observed in PDI and zeta potential compared to SpHL and SpHL-D; however, mean vesicle diameter values were significantly higher. Furthermore, the vesicular size, PDI, and zeta potential were not affected for the formulations containing both drugs, when compared to SpHL-S.

Regarding encapsulation efficiency, values of almost 100% and above 60% were obtained for DOX and SIM, respectively. For SIM, data evidenced that an increase in concentration did not result in a proportional drug-encapsulated increase, suggesting a possible saturation of the bilayer. Encapsulation efficiency at the theoretical concentration of 2 mg/mL was around 16% lower compared to 1 mg/mL. On the other hand, no significant difference was observed after DOX encapsulation for any of the formulations, demonstrating that SIM co-encapsulation did not alter the ability of the liposomal system to carry DOX. The molar ratio values calculated for formulations containing both drugs were equal to 1:1, 1:1.7, and 1:1.2 when the liposomes were prepared with 1 mg/mL of each drug, 1 mg/mL of DOX and 2 mg/mL of SIM, and 2 mg/mL of both drugs, respectively.

Based on these results, we defined the initial SIM concentration at 1 mg/mL to prepare all formulations containing the co-encapsulated drugs at molar ratios (DOX:SIM) 1:1, 1:2, and 2:1, respectively. These liposomes were further used for in vitro assays. The same physicochemical investigations were carried out (data are provided in Supplementary Material, Table S1) and there was no significant difference in the parameters evaluated compared to those shown in Table 1.

### 3.2. Cell Viability and Synergism Analysis

The cytotoxicity was investigated by SRB assay, and we screened for synergistic, additive effects, or antagonism between DOX and SIM, free or co-encapsulated, against different subtypes of human breast cancer cells.

All three cell lines tested (MDA-MB-231, SK-BR-3, and MCF-7) were sensitive to treatment with DOX and SIM. However, DOX showed higher cytotoxicity than SIM, as can be observed by the values of half-maximum inhibitory concentration (IC_50_) summarized in Table 2.

For the MDA-MB-231 strain, it was possible to observe that the combination of free DOX:SIM at the molar ratio of 2:1, respectively, was twice as cytotoxic as the DOX in monotherapy or combination therapy at an equimolar ratio. Furthermore, the drug association led to a significantly greater inhibitory effect than SIM monotherapy at all proposed ratios (1:1, 1:2, and 2:1). Encapsulation of DOX into pH-sensitive liposomes did not significantly alter its cytotoxicity against MDA-MB-231 compared to the free drug. Analysis of SpHL-D-S at molar ratios of 1:2 and 2:1 demonstrated more pronounced cytotoxic effects (about 2.1 and 2.4-fold, respectively) than SpHL-D.

The SK-BR-3 cell line was the most sensitive to treatment with DOX and SIM, as demonstrated by IC_50_ values lower than those obtained for MDA-MB-231. The combination of drugs did not increase the cytotoxic activity compared to DOX, either in the free or the co-encapsulated form for the SK-BR-3 cell line.

For the MCF-7 strain, lower sensitivity was detected compared to others, especially after treatments with liposomal formulations. In this cell line, there was no significant difference between the free DOX and the DOX:SIM combination in the free form for any of the molar ratios evaluated. However, co-encapsulation of DOX and SIM at 1:1 and 1:2, respectively, significantly reduced cytotoxic activity compared to SpHL-D. It is noteworthy that the control group (SpHL) had no effects on cell viability and was similar to untreated cells, indicating no significant toxicity of the formulation excipients [17] (data not shown ).

Taken together, the cell viability data suggest that the combination of DOX and SIM at a molar ratio of 2:1, respectively, either in the free form or co-encapsulated into liposomes, presented better results against the MDA-MB-231 cell line. However, no gains were observed against other cell lines.

**Figure 1:**
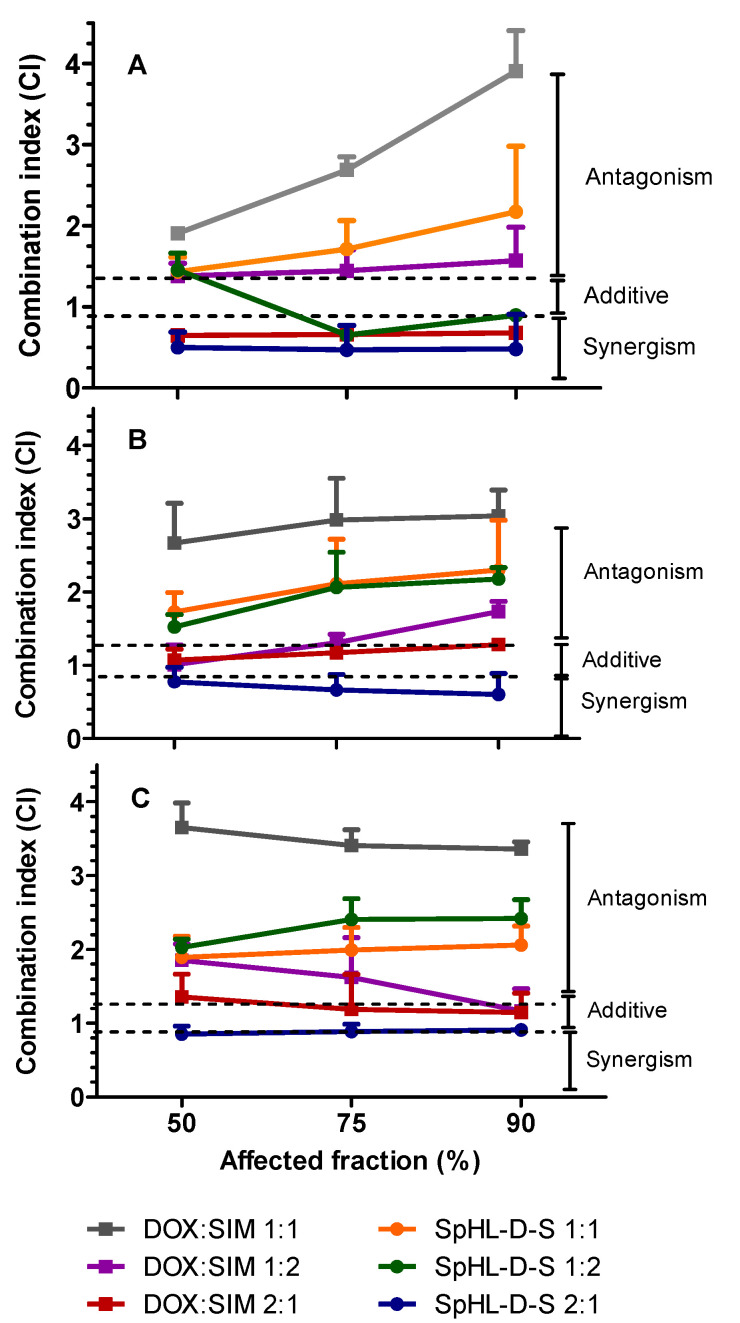
Fraction-affected X CI graph for free combinations and SpHL-D-S formulations in MDA-MB-231 (**A**), SK-BR-3 (**B**), and MCF-7 (**C**) cell lines. Note: All data are represented as mean ± SD (*n* = 3).

Figure 1 shows the combination indices (CI) for the different treatments against MDA-MB-231, SK-BR-3, and MCF-7 cell lines, in three inhibition concentrations (50, 75, and 90% of the cells). For MDA-MB-231, the combination of DOX:SIM at a ratio of 2:1, respectively, showed a potential synergistic effect for free and co-encapsulated forms (Figure 1A). The CI values for DOX:SIM 2:1 and SpHL-D-S 2:1 were, respectively, approximately 0.7 and 0.5 for all cellular inhibition concentrations analyzed. Furthermore, the treatment with SpHL-D-S 1:2 was partially synergistic, showing a CI close to 0.9 for the higher inhibition fractions (75 and 90%), while free DOX:SIM 1:2 treatment showed an additive effect.

A synergistic effect was also observed for SK-BR-3 after treatment with SpHL-D-S 2:1, showing CI between 0.6 and 0.8. On the other hand, the same molar ratio of free drugs resulted in an additive effect, with CI ranging from 1.0 to 1.3 (Figure 1B). Similar results were obtained for the MCF-7 cell line (Figure 1C). The association of drugs at 1:1, either free or encapsulated, led to antagonism (CI close to 2) for all cell lines investigated.

### 3.3. Nuclear Morphometric Analyses

Evaluation of NMA was based on an analytical tool developed by Filippi-Chiela and collaborators that allows for the extraction of morphometric data to classify nuclei into populations: normal (N), irregular (I), small and regular (SR), and large and regular (LR). The change in nuclear morphology can occur in processes associated with cell death. These modifications include the nuclear condensation and fragmentation observed in apoptosis, the nuclear size increase observed in senescence, and increases in nuclear irregularity under chemical or physical stresses [24].

**Figure 2:**
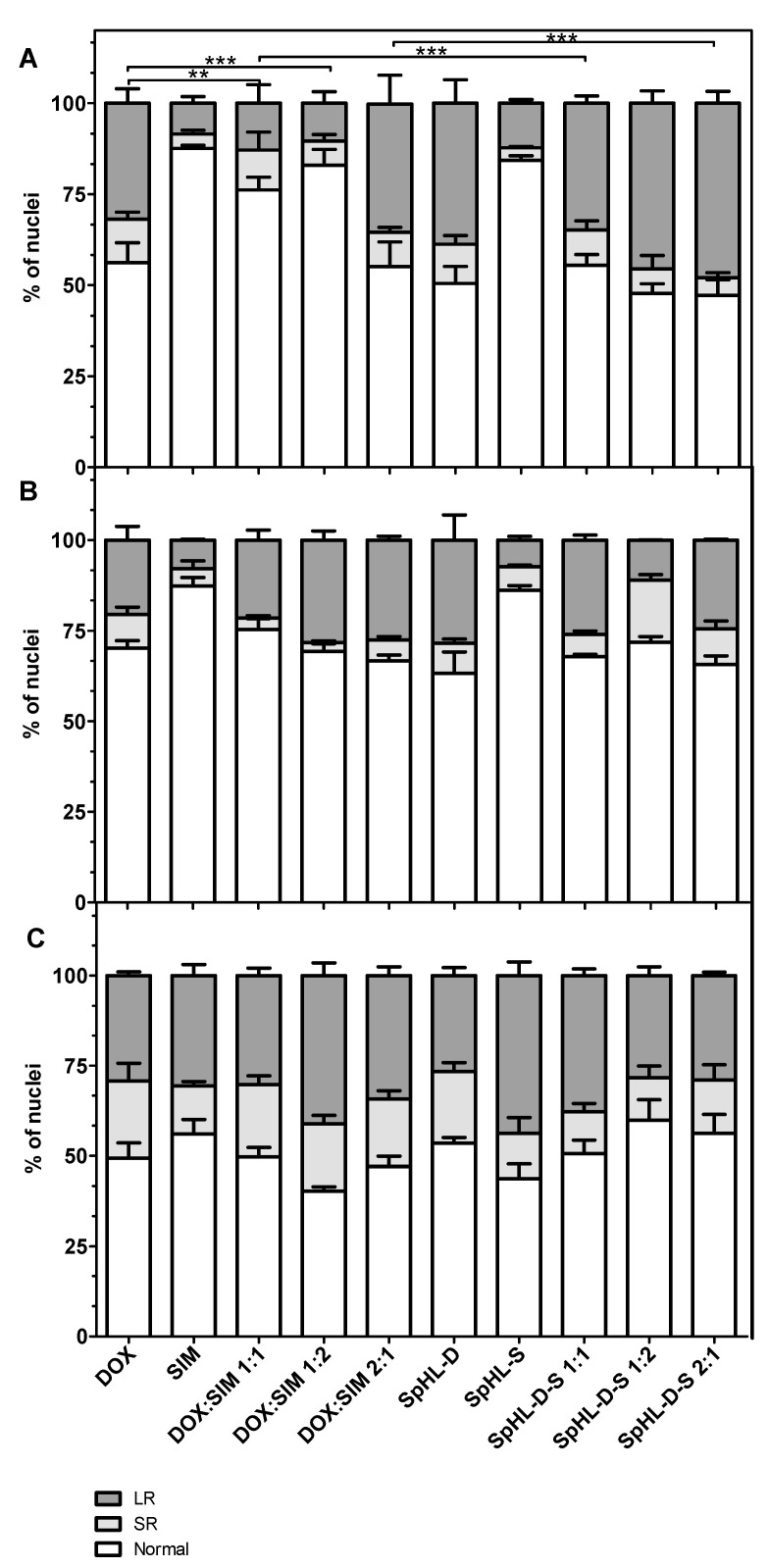
Nuclear morphometric distribution of cell lines (**A**) MDA-MB-231; (**B**) SK-BR-3, and (**C**) MCF-7, exposed to 80 nM of different treatments for 48 h. The bars represent normal (white bars), small and regular (light gray), and large and regular (dark gray) nuclei. Note: The data represent the mean ± SD of three independent experiments.; ** (*p* < 0.01) and *** (*p* < 0.001) represent a significant difference in relation to normal and LR nuclei (Bonferroni’s test).

The NMA data obtained after different treatments are shown in Figure 2. There was no significant number of irregular nuclei after different treatments for any of the cell lines. Furthermore, apoptosis events (SR nuclei) showed a similar extent for the three cell lines.

After DOX treatment, either in the free form or SpHL-D, MDA-MB-231 data analysis showed N nuclei ranging from 55 to 65%, SR equal to 10%, and LR varying from 25 to 35%. The values obtained after SIM treatment in both forms were 85%, 3%, and 12% for N, SR, and LR, respectively. These findings are in agreement with viability cell studies and reinforce the lower cytotoxic activity of SIM for this cell line. A significant reduction in LR nuclei was also verified, followed by increased N nuclei, after treatment with free DOX:SIM 1:1 or 1:2 compared to free DOX. In contrast, a significant increase in LR nuclei was detected after SpHL-D-S 1:2 and SpHL-D-S 2:1 treatments. It was also observed that the encapsulation of DOX:SIM 1:1 and 1:2 resulted in a significant increase (*p* < 0.001) in LR nuclei compared to free-form DOX at the same ratio. The increase in the levels of LR nuclei demonstrated an increase in senescence induction.

For SK-BR-3 cells, the percentage of distribution of nuclei was similar to MDA-MB-231 after SIM and DOX treatment. Furthermore, SIM treatment had a less pronounced cytotoxic effect compared to DOX treatments. No significant difference was observed between free or liposomal DOX, either alone or associated with SIM, for any of the molar ratios evaluated.

Concerning the MCF-7 cell line, no significant difference was detected between free DOX and other free forms or liposomal treatments. Similar to SK-BR-3, encapsulation of DOX:SIM at different molar ratios did not change the distribution of nuclei.

**Figure 3:**
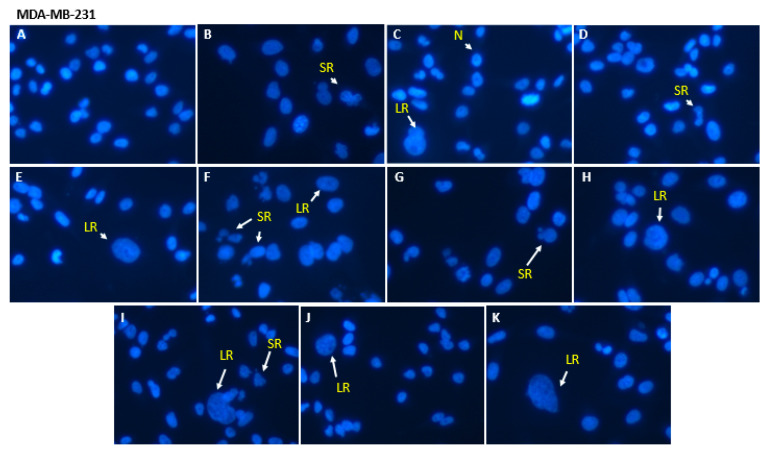
Representative fluorescence photomicrographs of breast cancer cell nuclei stained with Hoechst 33342 after treatments at a concentration of 80 nM, for 48 h: SpHL (**A**); free DOX (**B**); SIM free (**C**); DOX:SIM 1:1 (**D**); DOX:SIM 1:2 (**E**); DOX:SIM 2:1 (**F**) SpHL-D (**G**); SpHL-S (**H**); SpHL-D-S 1:1 (**I**); SpH-D-S 1:2 (**J**); or SpHL-D-S 2:1 (**K**). Note: Some of the different morphometric phenotypes of nuclei observed are indicated. N, normal; SR, apoptotic; LR, senescent. Images are representative of three independent experiments. Enhancement, 40×.

Figure 3 shows fluorescence photomicrographs of MDA-MB-231 nuclei stained with Hoescht 33342. An enlargement for cells exposed to the different treatments is evident compared to untreated cells. These characteristics correspond with the typical phenotypic morphology of senescence. It is also possible to observe the different distributions of LR and SR nuclei in relation to the control N that did not receive drug treatment. SK-BR-3 and MCF-7 cells had similar profiles to MDA-MB-231; thus, those images were not presented and are available in the Supplementary Materials (Figure S1).

### 3.4. Migration Assay

Cell migration was evaluated by a wound-healing assay that allowed the observation of two-dimensional cell migration in confluent monolayer cell cultures. SK-BR-3 human breast cancer is non-metastatic; in attempted invasive assays, this cell line did not show invasiveness, so it was not used at this stage of the study [25,26].

**Figure 4:**
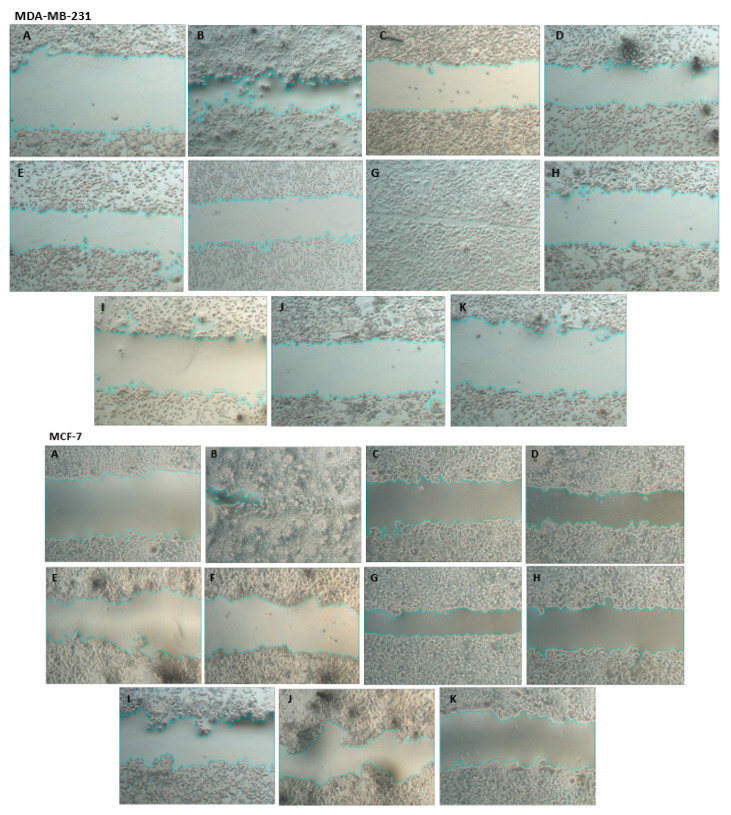
Representative phase contrast photomicrographs of MDA-MB-231 and MCF-7 cell lines exposed for 24 h at 80 nM to liposome treatments. Treatments: wound zero (control) (**A**); free DOX (**B**); SIM free (**C**); DOX:SIM 1:1 (**D**); DOX:SIM 1:2 (**E**); DOX:SIM 2:1 (**F**); SpHL-D (**G**); SpHL-S (**H**); SpHL-D-S 1:1 (**I**); SpH-D-S 1:2 (**J**); or SpHL-D-S 2:1 (**K**). 5× magnification.

Representative phase contrast photomicrographs of the scratches after 24 h of exposure to treatments are shown in Figure 4.

As can be seen in Table 3, free DOX or SpHL-D did not inhibit MDA-MB-231 or MCF-7 cell migration. However, all treatments containing SIM significantly reduced the percentage of cell migration compared to treatments containing only DOX for both cell lines, except the DOX:SIM 1:1 treatment. For the MDA-MB-231 strain, it was observed that the combined treatments DOX:SIM 1:2 and 2:1 in the free form inhibited, respectively, two- and three-fold more cell migration compared to free DOX. On the other hand, SpHL-D-S therapy at a 1:2 molar ratio was about three-fold more inhibitory, and at a 2:1 ratio, about six-fold more inhibitory, when compared to SpHL-D. A similar profile was obtained for MCF-7. The combined treatments DOX:SIM 1:2 and 2:1 were, respectively, four and three times more inhibitory to migration compared to free DOX, while SpHL-D-S 1:2 and 2:1 inhibited two and three times more than SpHL-D.

The in vitro studies clearly showed the difference in the response of cell lines to treatments. Furthermore, they were important as a screening to select the best molar ratio for DOX and SIM in order to proceed with formulation characterizations and guide future investigations. As previously reported, treatment with SpHL-D-S 2:1 showed a synergistic effect in all fractions and against all human breast tumor cell lines evaluated, so this ratio was used for a more detailed characterization of stability and morphological evaluation.

### 3.5. Drug Release Study

**Figure 5:**
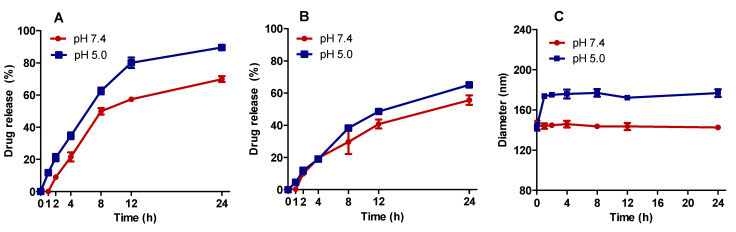
DOX (**A**) and SIM (**B**) release profiles from SpHL-D-S at pH 7.4 (red) and 5.0 (blue) and evaluation of vesicle diameter (**C**) at different times.

The DOX and SIM release profile from SpHL-D-S (2:1) was evaluated using a dialysis method at two different pH levels (7.4 and 5.0). Before the release study, various release conditions were tested to determine the sink conditions for both active substances (data not presented), and the HEPES buffer plus Tween 80 (0.1% *w*/*v*) was used. As shown in Figure 5, drug release was pH- and time-dependent. SpHL-D-S incubated at pH 5.0 showed higher DOX and SIM release than at pH 7.4. After 24 h of incubation, the DOX release was around 90% and 70% at pH 5.0 and 7.4, respectively. SIM release was more controlled, and near 65% and 56% was released after 24 h of incubation at pH 5.0 and 7.4.

At pH 7.4, the vesicle size was not changed for 24 h. In contrast, significant changes in the vesicle diameter were noted at pH 5.0 from 1 h of evaluation (Figure 5C), since the diameter of the vesicles increased by around 23% (142.6 ± 1.9 nm versus 176.7 ± 3.1 nm). The increase in vesicle size is also indicative that the SpHL-D-S responds to pH variation, since the low pH leads to vesicle aggregation and/or membrane fusion [16].

### 3.6. Cryo-TEM

**Figure 6:**
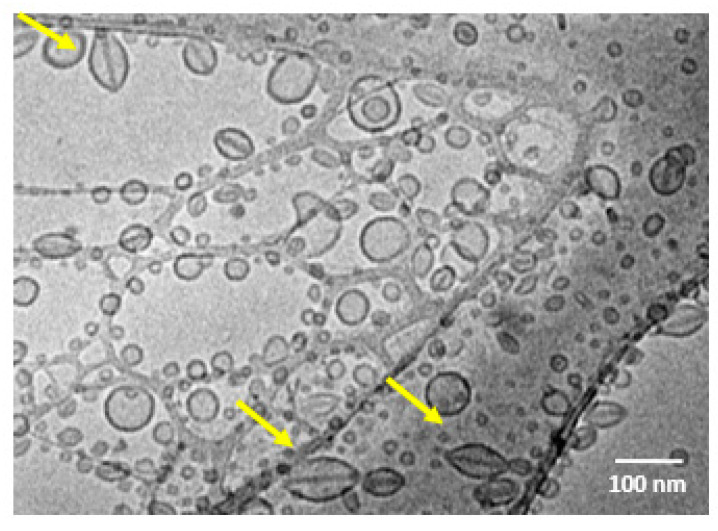
Cryo-TEM photomicrograph of SpHL-D-L 2:1. Note: Yellow arrows indicate DOX sulfate crystals within the liposomes.

Morphological analysis of SpHL-D-S at a molar ratio of 2:1 was also performed by cryo-TEM (Figure 6). Images showed the presence of vesicles which were spherical and non-spherical, unilamellar, and reasonably uniform in diameter. The non-spherical form can be attributed to the presence of DOX crystals that force a change in the shape of the vesicles from spherical to non-spherical [27].

### 3.7. Storage Stability

**Figure 7:**
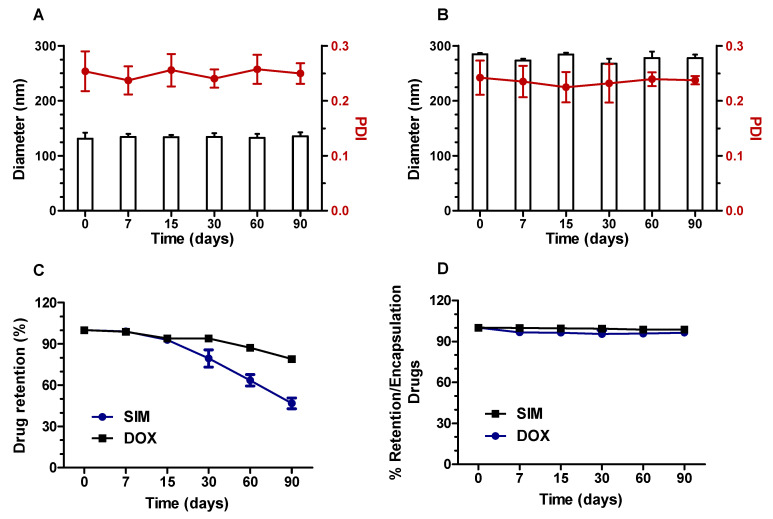
Average size, PDI, and % retention of drugs of SpHL-D-S 2:1, kept at 4 °C in liquid form (**A**,**C**) or prepared by reconstitution of lyophilized SpHL-S (**B**,**D**) and evaluated over 90 days.

The physicochemical stability of SpHL-D-S 2:1 was studied over 90 days. No notable changes were observed in vesicle size and PDI (Figure 7A) for at least 90 days at 4 °C. Regarding the DOX and SIM retention into liposomes, both drugs were stable during the first 15 days. However, from the 15th day onwards, a gradual and significant reduction in the SIM-encapsulated level was observed, reaching about 50% in 90 days. The DOX concentration was maintained over time.

The reduction in the content of SIM may be due to its sensitivity to the aqueous solution. As SIM contains a lactone ring labile to hydrolysis, in an aqueous medium, hydrolysis may take place, resulting in a compound with lower lipophilicity. To avoid hydrolysis, SpHL-S was lyophilized and stored in its freeze-dried form for reconstitution with DOX at the moment of use. The results are presented in Figure 7B,D. Larger diameters (around 280 nm) with PDI near 0.25 were obtained after the lyophilization process using glucose as a lyoprotectant, and these values also remained unchanged for at least 90 days. Furthermore, the SIM content and the DOX encapsulation capacity remained close to 100% for at least 90 days (Figure 7D).

## 4. Discussion

Although chemotherapy has played a central role in breast cancer treatment, the ability of drugs to kill cancer cells with minimal damage to healthy tissue is still a challenge, being one of the main requirements for the success of cancer therapy. It is well-described that most chemotherapeutic agents, such as DOX, may cause adverse effects that are potentially fatal to the patients. The most relevant, and sometimes irreversible, toxic effect of DOX is cardiomyopathy. To improve the safety profile of this antineoplastic drug, encapsulation into liposomes has been considered a promising alternative since it can promote an increase in drug selectivity, as it preferentially accumulates in the target tissue, reducing damage to healthy areas. Doxil^®^, the first liposomal formulation approved for breast cancer treatment, presented reduced cardiotoxicity and myelosuppression induced by DOX. Despite these advantages, there are still reports of cardiac toxicity in 11% of patients treated with this medication. Our research group has shown that pH-sensitive liposomes composed of DOPE, CHEMS, and DSPE-PEG carrying DOX and associated with other substances with antitumor potential are beneficial compared to liposomal formulations similar to Doxil^®^ [28,29]. Recent studies have also reported that SIM exhibits anticancer activity since it can influence proliferation, migration, and cancer cell survival, and there are indications of protective factors during cancer chemotherapy in patients using SIM [30]. Therefore, we proposed that the potential of SIM to increase the DOX antitumor activity after short-term exposure of the breast cancer cell lines to treatment be investigated.

The first step of the study consisted of formulation development with suitable physicochemical properties for biological evaluation. All formulations showed homogeneity and small size (lower than 150 nm), despite the size having been increased by SIM presence (Table 1). This fact might be due to the hydrophobic nature of SIM, which favors its interaction with the lipid bilayer and increases the size of the vesicles [21,31]. It has been reported that small particle size and narrow size distribution are quality attributes of liposome drug products, especially for an injectable formulation, besides allowing the efficient delivery of antitumor agents to a tumor by passive targeting. These parameters did not change over time, as can be seen in Figure 7A. Furthermore, zeta potential values near the neutral range were obtained for all formulations, and can be attributed to the low electrophoretic mobility caused by the hydrodynamic resistance of the PEG molecules coupled to the DSPE-PEG2000 [21]. Previous studies have demonstrated that nanoparticle surfaces with no charge bind less protein than those that are negatively or positively charged, increasing blood circulation time [32].

As regards encapsulation efficiency, higher values were observed for SIM (>60%) and DOX (almost 100%). The high encapsulation obtained by the DOX active loading is often explained by the formation of insoluble DOX-sulfate crystals inside the aqueous core of liposomes [33]. These crystals, previously named “coffee bean,” could be clearly observed by cryo-TEM (Figure 6). Regarding SIM, we suggest that the unsaturated phospholipid (DOPE) present in the formulation favored the “pockets” formation in the bilayer, in which the hydrophobic molecule as SIM was embedded. However, the SIM retention efficiency decreased over time (Figure 7C), likely due to the SIM hydrolysis in a more hydrophilic compound with less affinity by bilayer. To overcome this drawback, the formulation was lyophilized. The dry product was able to be stored for a long time and hydrated immediately before use [34]. After that, the concentration of both drugs was kept near 100% for 90 days. The mean diameter increased by around two-fold compared to liquid form. It has been reported that the lyophilization process, even with the use of cryoprotectants, can cause stress to the liposomal vesicles. In addition, at the time of reconstitution, the particles can aggregate, generating a larger diameter, as seen in Figure 5B [35].

It is well-known that the antitumor efficacy of pH-sensitive liposomes depends on their ability to release the drug into the tumor region. In an acidic environment, these formulations, composed of DOPE (a fusogenic lipid), can fuse or destabilize, releasing the encapsulated content [36,37]. Herein, the release study was carried out at pH 5.0 and 7.4. The release profile showed that the released total percentage of both drugs was higher at pH 5.0 than 7.4 (Figure 5). In addition, there was a significant change in the diameter of vesicles at pH 5.0. These data suggest the pH sensitivity property of the system, even with the addition of SIM to the lipid bilayer. Furthermore, assuming that the pH in the non-tumor environment were 7.4, most of the drugs would remain circulating retained into the liposomes for a longer time and, thus, could lead to less toxicity in normal tissues [22,38].

As breast cancer shows multiple subtypes with histopathological and biological differences that can lead to different treatment responses, we have chosen to evaluate the behavior of the formulations in two luminal subtype cell lines, namely MCF-7 and SK-BR-3, and one basal subtype cell line, MDA-MB-231. The first one is positive for estrogen receptors (ER) and progesterone receptors (PR) and negative for human epidermal growth factor receptor 2 (HER2). The second is ER-/PR-/HER2+, and the last is triple-negative breast cancer (ER–/PR–/HER2) [39].

The cytotoxicity assessment showed more pronounced sensitivity for SK-BR-3, followed by MDA-MB-231 and MCF-7, especially after DOX: SIM 2:1 treatment in free or co-encapsulated form. In addition, SpHL-D-S at the molar ratio of 2:1 presented a synergistic effect for all lines tested. Considering that the potential cytotoxic activity of SIM is related to its ability to modulate some effects on reactive oxygen species (ROS), deregulate caspase cascades, and inhibit 3-hydroxy-3methyl-glutaryl-coenzyme A reductase (overexpressed in cancer cells) [40,41,42,43,44], while the DOX mechanism of action refers to intercalation in DNA and interruption of DNA repair mediated by topoisomerase II [8], we may suggest that these different mechanisms favor the possibilities of synergism and increase the sensitivity of cells, thus minimizing the development of resistance [45]. This hypothesis is supported by previous studies that have reported that combined DOX and SIM therapy significantly stopped the growth of prostate cancer cells through multiple mechanisms such as increased levels of intracellular ROS, induced apoptosis, promoted cellular autophagy, and anti-angiogenesis [46]. Buranrat et al. reported that the combination of DOX and SIM increased cytochrome c protein expression and caspase-3 activity compared to each drug alone, suggesting that SIM sensitizes MCF-7 breast tumor cells, potentiating the action of DOX [47]. Furthermore, Machado and coworkers suggested that MCF-7 cells are more resistant to oxidative damage caused by ROS compared to MDA-MB-231 cells, thus suffering less apoptosis [48]. This fact could explain the results obtained herein, in which IC50 values for MCF-7 were higher than MDA-MB-231 cells, especially after treatment with liposomal formulations.

## 5. Conclusions

In this study, we developed a novel formulation of pH-sensitive liposomes containing DOX and SIM. Our results show that this system is pH-responsive and stable in the lyophilized form for at least 90 days. The viability, CI, and NMA data pointed out that the 2:1 molar ratio can act synergistically, improving the inhibitory results of proliferation and induction of death of breast cancer cells. Furthermore, the migration results reinforce the notion that the combination of DOX and SIM significantly improves the inhibition of cell proliferation. However, further studies are needed to understand the molecular mechanisms involved.

In summary, the present study identified a new strategy for a potential combination therapy. SpHL-D-L 2:1 showed suitable physicochemical properties, release behaviors, and cytotoxicity responses to be considered a promising alternative for further in vivo breast cancer therapy.

## Figures and Tables

**Table 1:** Physicochemical characteristics (mean diameter, PDI, zeta potential, encapsulation percentage—EP, and drug concentration) for the different liposomal formulations containing individual or co-encapsulated drugs.

Formulations	[Drug] Theoretical (mg/mL)		Mean Diameter (nm)	PDI	Zeta Potential (mV)	[Drug] Experimental (mg/mL)		EP (%)		Molar Ratio DOX:SIM
	DOX	SIM				DOX	SIM	DOX	SIM	
SpHL	0	0	113 ± 9.0	0.07 ± 0.03	−3.5 ± 0.80	-	-			
SpHL-D	0	1	123 ± 5.0	0.12 ± 0.03	−3.6 ± 0.90	0.99 ± 0.02	-	99 ± 0.1	-	-
SpHL-S	0	1	139 ± 2.6 ^a^	0.22 ± 0.02	−3.72 ± 0.17	-	0.76 ± 0.06	-	76 ± 6	-
SpHL-D-S	1	1	145 ± 3.7 ^a^	0.23 ± 0.03	−3.63 ± 0.14	0.98 ± 0.01	0.74 ± 0.05	98 ± 0.5	74 ± 0.5	1:1
	1	2	140 ± 1.1 ^a^	0.12 ± 0.06	−3.39 ± 0.44	0.97 ± 0.01	1.26 ± 0.02	98 ± 0.5	63 ± 1.2	1:1.7
	2	2	147 ± 1.6 ^a^	0.21 ± 0.02	−3.33 ± 0.32	1.97 ± 0.01	1.28 ± 0.02	99 ± 0.3	64 ± 0.7	1.2:1

^a^ Represents a significant difference from the formulation containing SpHL and SpHL-D. Data are expressed as mean ± standard deviation (SD). *n* = 3.

**Table 2:** IC_50_ values obtained for the breast cell lines exposed for 48 h to different proportions of DOX and SIM in free form or co-encapsulated into liposomes.

Treatments		IC_50_ (µM)	
	MDA-MB-231	SK-BR-3	MCF-7
DOX	0.80 ± 0.19	0.20 ± 0.07	0.71 ± 0.14
SIM	1.53 ± 0.37 ^a^	0.85 ± 0.19 ^a^	1.95 ± 0.63 ^a^
DOX:SIM (1:1)	0.96 ± 0.27 ^b,d^	0.33 ± 0.07 ^b^	1.15 ± 0.33 ^b^
DOX:SIM (1:2)	0.71 ± 0.17 ^b^	0.19 ± 0.05 ^b^	0.91± 0.23 ^b^
DOX:SIM (2:1)	0.44 ± 0.07 ^a,b^	0.19 ± 0.05 ^b^	0.99 ± 0.35 ^b^
SpHL-DOX	0.73 ± 0.14	0.32 ± 0.09	1.03 ± 0.32
SpHL-SIM	0.86 ± 0.23	0.83 ± 0.19 ^c^	4.28 ± 0.99 ^c^
SpHL-D-S (1:1)	0.47 ± 0.24	0.35 ± 0.06	3.98 ± 0.87 ^c,e^
SpHL-D-S (1:2)	0.35 ± 0.12 ^c^	0.35 ± 0.05	2.39 ± 1.07 ^c,e^
SpHL-D-S (2:1)	0.31 ± 0.08 ^c^	0.23 ± 0.07	0.87 ± 0.34

Data are expressed as mean ± DP. Letters represent significant differences compared to: ^a^ free DOX, ^b^ free SIM, ^c^ SpHL-D, ^d^ free DOX:SIM (2:1),^e^ free form at the same molar ratio. *p* < 0.05 was considered a significant difference (Tukey’s test).

**Table 3:** Percentage of cell migration in relation to control for MDA-MB-231 and MCF-7 cell lines evaluated after exposure to the free or encapsulated drugs at different molar ratios.

Treatments	MDA-MB-231	MCF-7
DOX	89.8 ± 6.0	90.1 ± 6.0
SIM	39.7 ± 5.0 ^a^	37.9 ± 4.3 ^a^
DOX:SIM (1:1)	47.9 ± 10.5 ^c^	49.2 ± 3.7
DOX:SIM (1:2)	42.1 ± 6.7 ^a^	22.0 ± 7.8 ^a^
DOX:SIM (2:1)	27.8 ± 4.2 ^a^	28.3 ± 8.1 ^a^
SpHL-DOX	90.6 ± 5.8	87.9 ± 3.0
SpHL-SIM	24.5 ± 2.4 ^b^	32.6 ± 8.8 ^b^
SpHL-D-S (1:1)	25.2 ± 2.2 ^b^	41.9 ± 5.4 ^b^
SpHL-D-S (1:2)	24.8 ± 4.6 ^b^	42.9 ± 1.3 ^b^
SpHL-D-S (2:1)	15.5 ± 5.7 ^b^	24.7 ± 2.2 ^b^

^a^ significant difference compared to treatment with free DOX; ^b^ significantly different from SpHL-D treatment; ^c^ significant difference compared to treatment with DOX:SIM 2:1. Data for cell lines MDA-MB-231 and MCF-7 were transformed as y = log(value + 1). A significant difference was considered for *p*-values < 0.05 (Tukey’s test).

## Data Availability

Not applicable.

## Supplementary Materials

The following supporting information can be downloaded at: https://www.mdpi.com/article/10.3390/pharmaceutics15020369/s1, Figure S1: Representative fluorescence photomicrographs of breast cancer cell nuclei stained with Hoechst 33342 after treatments at a concentration of 80 nM, for 48 h: SpHL (A); free DOX (B); SIM free (C); DOX:SIM 1:1 (D); DOX:SIM 1:2 (E); DOX:SIM 2:1 (F) SpHL-D (G); SpHL-S (H); SpHL-D-S 1:1 (I); SpH-D-S 1:2 (J) or SpHL-D-S 2:1 (L); Table S1: Physicochemical characteristics for the different formulations.
