# Supplementary material for: Co-Encapsulation of Simvastatin and Doxorubicin into pH-Sensitive Liposomes Enhances Antitumoral Activity in Breast Cancer Cell Lines
Source: Pharmaceutics. 2023 Jan 21;15(2):369. doi: 10.3390/pharmaceutics15020369 (PMC9960841; doi:10.3390/pharmaceutics15020369)
Supplement: Supplementary file 1 [file pharmaceutics-15-00369-s001.zip › pharmaceutics-2131978-supplementary.pdf]

pharmaceutics

Supplementary Materials: Co-Encapsulation of Simvastatin and Doxorubicin into pH-Sensitive Liposomes Enhances Antitumoral Activity in Breast Cancer Cell Lines

Jaqueline Aparecida Duarte, Eliza Rocha Gomes, Andre Luis Branco de Barros and Elaine Amaral Leite

Table S1. Physicochemical characteristics for the different formulations.

Molar ratio SpHL-D-S	Mean diameter (nm)	PDI	Zeta potential (mV)	Encapsulated drug concentration (mg/mL) [DOX] (mg/mL)	[SIM] (mg/mL)
1:1	139 ± 2.6	0.22 ± 0.03	−3.39 ± 0.28	0.93 ± 0.06	0.72 ± 0.05
1:2	140 ± 1.3	0.19 ± 0.02	−3.56 ± 0.55	0.50 ± 0.07	0.76 ± 0.04
2:1	136 ± 6.9	0.21 ± 0.04	−3.68 ± 0.76	1.97 ± 0.11	0.77 ± 0.10

Data are expressed as mean ± standard deviation (SD). n = 3.

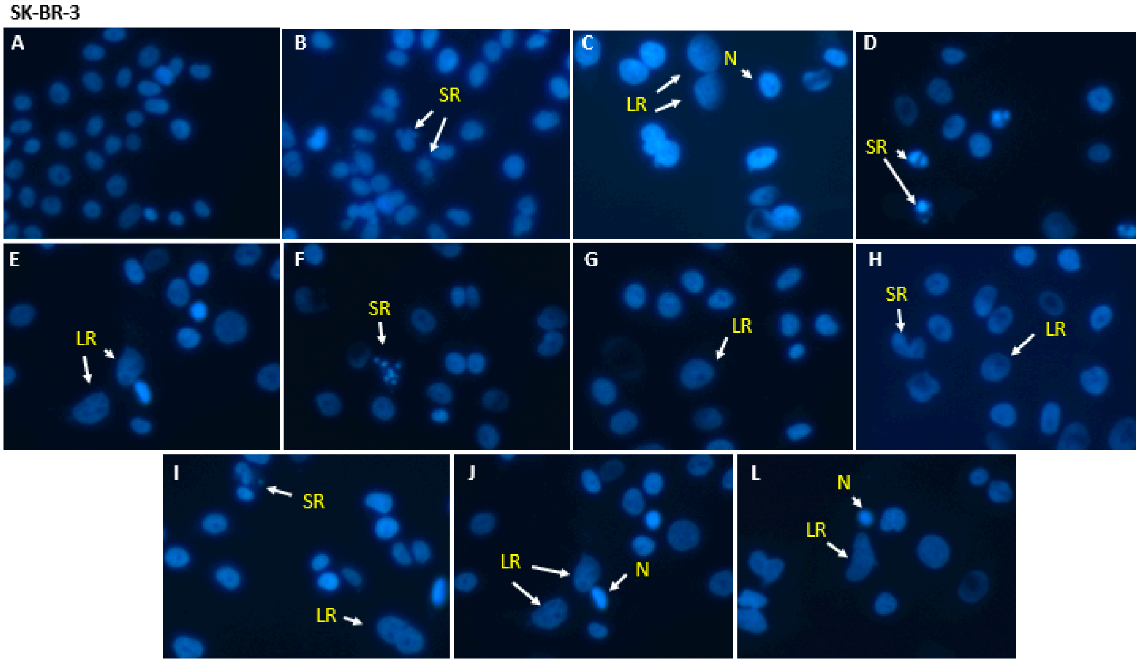

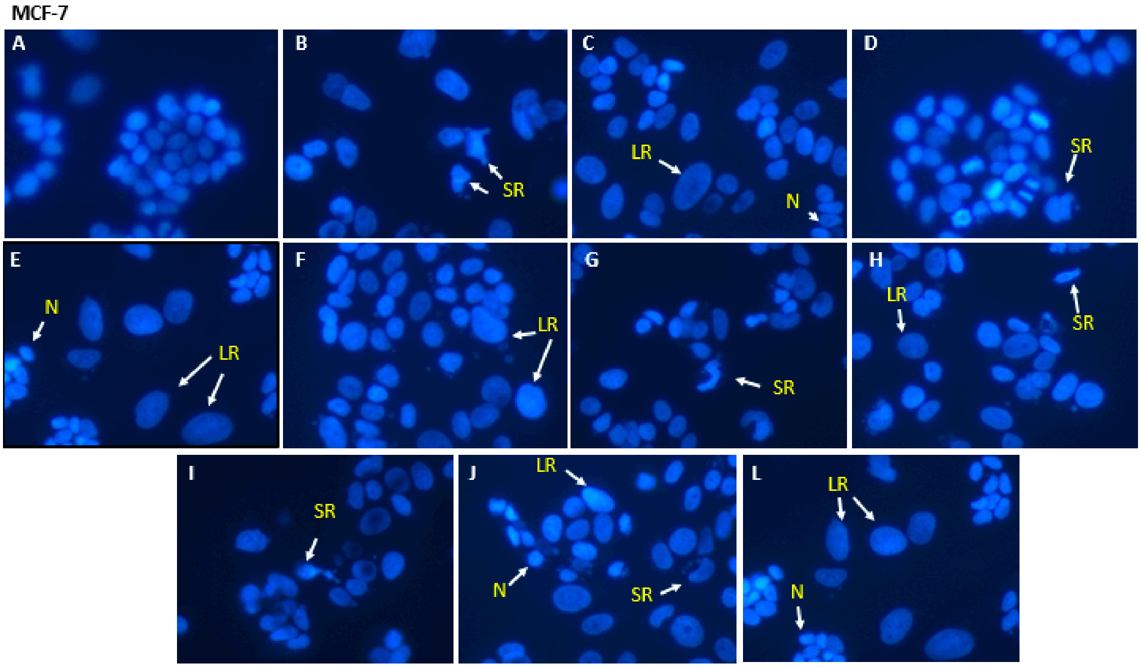

Figure S1. Representative fluorescence photomicrographs of breast cancer cell nuclei stained with Hoechst 33342 after treatments at a concentration of 80 nM, for 48 h: SpHL (**A**); free DOX (B); SIM free (C); DOX:SIM 1:1 (**D**); DOX:SIM 1:2 (**E**); DOX:SIM 2:1 (**F**) SpHL-D (**G**); SpHL-S (**H**); SpHL-D-S 1:1 (**I**); SpH-D-S 1:2 (**J**) or SpHL-D-S 2:1 (**L**).
